# Temporal Dynamics of Emerging Cardiovascular Biomarkers in the Early Phase After Acute ST-Segment Elevation Myocardial Infarction: A Prospective Single-Center Pilot Study

**DOI:** 10.3390/cells15201868

**Published:** 2026-10-14

**Authors:** Stefan S. Zivkovic, Vojko V. Misevic, Kosta S. Krupnikovic, Aleksa D. Obradovic, Milos D. Babic, Bojana D. Saroskovic, Andjelka M. Isakovic, Vedrana R. Pavlovic, Milica N. Dekleva, Goran M. Loncar, Milan R. Dobric, Petar M. Otasevic, Milovan M. Bojic, Gordana G. Mihajlovic

**Affiliations:** 1 Clinic for Cardiology, National Institute for Heart and Blood Vessels “Dedinje”, 11000 Belgrade, Serbia; misevic.vojko@gmail.com (V.V.M.); krupnikovick@gmail.com (K.S.K.); obradovicaleksa96@gmail.com (A.D.O.); babicmisa@hotmail.com (M.D.B.); loncar_goran@yahoo.com (G.M.L.); iatros007@gmail.com (M.R.D.); potasevic@yahoo.com (P.M.O.); milovan.bojic@institutdedinje.org (M.M.B.); 2 Institute for Cardiovascular Diseases of Vojvodina, 21204 Sremska Kamenica, Serbia; bojana.saroskovic@gmail.com; 3 Institute of Medical and Clinical Biochemistry, Faculty of Medicine, University of Belgrade, Pasterova 2, 11000 Belgrade, Serbia; andjelka.isakovic@med.bg.ac.rs; 4 Institute of Medical Statistics and Informatics, Faculty of Medicine, University of Belgrade, 11000 Belgrade, Serbia; vedrana.pavlovic@med.bg.ac.rs; 5 Faculty of Medicine, University of Belgrade, 11000 Belgrade, Serbia; dekleva.milica@gmail.com (M.N.D.); goca.mihajlovic@yahoo.com (G.G.M.); 6 “Zvezdara” University Medical Center, 11000 Belgrade, Serbia

**Keywords:** acute ST-elevation myocardial infarction, primary percutaneous coronary intervention, biomarker kinetics, soluble suppression of tumorigenicity-2, galectin-3, mid-regional pro-adrenomedullin, growth differentiation factor 15, left ventricular remodeling

## Abstract

**Highlights:**

**What are the main findings?**

- Soluble suppression of tumorigenicity-2 (sST2) and mid-regional pro-adrenomedullin (MR-proADM) showed distinct early changes after ST-segment elevation myocardial infarction (STEMI), with the highest measured concentrations at 12 and 24 h, respectively, while no significant change over time was found for galectin-3 (Gal-3) and growth differentiation factor 15 (GDF-15).
- In exploratory analyses, higher sST2 and MR-proADM concentrations were observed in patients with left ventricular ejection fraction below 40%, and higher sST2 concentrations were found in patients with left anterior descending artery infarction.

**What are the implications of the main findings?**

- The distinct kinetics of sST2 and MR-proADM suggest potentially informative biomarker-specific sampling windows for future prognostic assessment after STEMI.
- Larger prospective studies are needed to validate these temporal patterns and determine their prognostic value.

**Abstract:**

Introduction: Acute ST-segment elevation myocardial infarction (STEMI) remains a major cause of cardiovascular morbidity and mortality. Despite successful primary percutaneous coronary intervention (pPCI), early myocardial injury and stress may contribute to left ventricular dysfunction. The optimal timing of biomarker assessment after STEMI remains uncertain. Objective: To characterize the early kinetics of soluble suppression of tumorigenicity-2 (sST2), galectin-3 (Gal-3), mid-regional pro-adrenomedullin (MR-proADM), and growth differentiation factor 15 (GDF-15) during the first 96 h after STEMI and identify potentially informative sampling time points for future prognostic assessment. Secondary, exploratory objectives were to evaluate differences in biomarker concentrations according to symptom duration, left ventricular ejection fraction (LVEF), and infarct-related artery. Methods: In this prospective, single-center, exploratory pilot study, patients aged 18–75 years with first STEMI treated with pPCI within 6 h of symptom onset were enrolled between September 2024 and November 2025. Blood samples were collected at admission and 12 ± 4, 24 ± 4, 48 ± 8, and 96 ± 8 h after pPCI. Biomarkers were measured by ELISA, with high-sensitivity cardiac troponin I (hs-cTnI) as a reference marker of myocardial injury. Results: Of 46 enrolled patients, 16 were excluded, mostly due to early discharge, and 30 patients with complete samples at all five time points were included in the analysis (mean age 57.9 ± 11.1 years; 66.7% male). sST2, MR-proADM, and hs-cTnI showed significant temporal variation (all *p* < 0.001), with the highest measured concentrations of sST2 at 12 h and of MR-proADM at 24 h. No significant change over time was found for Gal-3 and GDF-15. In exploratory analyses, sST2 and MR-proADM concentrations were higher in patients with LVEF < 40%, and sST2 concentrations were higher in patients with left anterior descending artery (LAD) infarction. Symptom duration was not associated with biomarker concentrations. Conclusions: sST2 and MR-proADM show distinct early post-STEMI kinetics, suggesting potentially informative sampling windows for future prognostic studies. Larger prospective studies are needed to confirm these kinetics and the exploratory differences according to left ventricular function and infarct location, and to evaluate the prognostic value of these sampling windows with echocardiographic and clinical follow-up.

## 1. Introduction

Acute ST-segment elevation myocardial infarction (STEMI) continues to be a major cause of acute cardiovascular morbidity and mortality worldwide. Although its incidence has declined in developed countries due to improved primary prevention, risk factor control, and early recognition of ischemic symptoms, STEMI still contributes substantially to hospital admissions and poses a considerable long-term cardiovascular risk, especially among older adults and those with comorbidities [1,2].

Advances in acute management, particularly primary percutaneous coronary intervention (pPCI) combined with optimized pharmacological therapy, have markedly improved short-term survival after myocardial infarction (MI). Contemporary reperfusion strategies, intensive care, and antithrombotic regimens have significantly reduced both intrahospital and one-year mortality in recent cohorts [3,4,5]. Despite these improvements, survivors remain susceptible to serious complications, most notably left ventricular dysfunction and heart failure, which emerge predominantly during the first year following the infarction [6,7].

Adverse left ventricular remodeling plays a central role in post-MI adaptation and is a key determinant in the development of heart failure. This complex process involves structural, functional, and molecular alterations, including changes in ventricular geometry, wall thickness, and myocardial composition. Mechanical load, neurohormonal activation, inflammatory signaling, and extracellular matrix turnover all contribute to remodeling [8,9]. A substantial proportion of patients experience significant adverse remodeling within the first months after MI, with larger infarcts, particularly anterior, carrying the highest risk for maladaptive changes [10,11]. Infarct size, ongoing ischemia, hemodynamic stress, neurohormonal activation, and comorbidities such as diabetes and obesity contribute to adverse remodeling, whereas timely reperfusion and optimized medical therapy promote reverse remodeling and improved clinical outcomes [12,13].

Over the past decade, an increasing number of circulating biomarkers have been investigated as potential markers of early post-MI remodeling [14,15,16]. High-sensitivity assays and advances in molecular biology have enabled the identification of markers reflecting myocardial stress, inflammation, fibrosis, oxidative stress, and extracellular matrix turnover [17,18]. Multimarker strategies are increasingly emphasized, as they can capture the complex, overlapping processes of myocardial injury and early repair more effectively than single-marker approaches [19,20].

Among the most extensively studied biomarkers are soluble suppression of tumorigenicity-2 (sST2), galectin-3 (Gal-3), mid-regional pro-adrenomedullin (MR-proADM), and growth differentiation factor 15 (GDF-15).

sST2, a member of the interleukin-1 receptor family, exists in both transmembrane (ST2L) and soluble (sST2) forms. IL-33 binding to ST2L exerts cardioprotective effects, whereas circulating sST2 acts as a decoy receptor, inhibiting IL-33/ST2L signaling. Elevated sST2 levels are linked to inflammation, fibrosis, maladaptive remodeling, and increased mortality in acute and chronic heart failure as well as in stable coronary artery disease [21,22,23].

Gal-3 is a β-galactoside-binding lectin secreted mainly by activated macrophages. It promotes fibroblast activation, collagen deposition, and extracellular matrix remodeling. Elevated Gal-3 concentrations are associated with myocardial fibrosis, adverse ventricular remodeling, and increased cardiovascular and all-cause mortality in both heart failure with reduced—HFrEF and preserved left ventricular ejection fraction (LVEF)—HFpEF. Because of its relative stability and association with tissue fibrosis, Gal-3 has been proposed for early risk stratification, although its additional clinical value remains uncertain [24,25,26].

MR-proADM, a stable fragment of adrenomedullin, reflects vasodilation, natriuresis, and cardiovascular stress. Circulating MR-proADM levels provide prognostic information in acute myocardial infarction and early post-infarction heart failure, independently predicting mortality, rehospitalization, and adverse cardiovascular events, often adding value beyond BNP and NT-proBNP [27,28].

GDF-15, a stress-responsive cytokine of the transforming growth factor-β superfamily, is upregulated in response to inflammation, oxidative stress, and myocardial injury. Elevated GDF-15 concentrations are associated with increased risk of heart failure, adverse early remodeling, and worsening functional status, regardless of baseline LVEF. It complements traditional biomarkers in identifying high-risk patients in the immediate post-infarction period [29,30,31].

Together, sST2, Gal-3, MR-proADM, and GDF-15 provide a multidimensional view of myocardial stress, inflammation, fibrosis, and remodeling, reflecting distinct but overlapping pathophysiological pathways in the early post-MI period.

Despite extensive research on individual biomarkers, the optimal timing for blood sampling to capture the most informative levels remains unclear. Most studies rely on single measurements, reflecting only a fixed point of dynamic biological processes. Systematic assessment of serial biomarker trajectories across the early post-MI period could offer a more complete understanding of their fluctuations and temporal patterns. Such an approach may identify periods when biomarker concentrations are most informative, which could help in the design of future studies of post-infarction remodeling and clinical outcomes.

Therefore, this study aimed primarily to characterize the temporal dynamics of sST2, Gal-3, MR-proADM, and GDF-15 during the first 96 h following STEMI treated with pPCI and to identify the time points at which their circulating concentrations may be most informative for future prognostic assessment. Secondary, exploratory aims were to evaluate whether biomarker trajectories differ according to symptom duration, left ventricular systolic function, and infarct-related artery.

## 2. Materials and Methods

### 2.1. Study Population

This prospective cohort study was conducted at the National Institute for Heart and Blood Vessels “Dedinje”, Belgrade, Serbia, between September 2024 and November 2025. The study protocol was approved by the Ethics Committee of the Institute “Dedinje” (approval numbers: 4671, date 16 September 2024, original protocol; and 1345, date 13 March 2026. amendment for sST2 analysis) and was performed in accordance with the Declaration of Helsinki.

Patients aged 18–75 years presenting with a first STEMI and treated with pPCI within six hours of symptom onset were eligible. Exclusion criteria included a history of coronary artery disease (prior myocardial infarction, unstable or stable angina, documented significant coronary artery stenosis, or previous revascularization), chronic heart failure, pre-procedural recanalization of the infarct-related artery, chronic total occlusion or critical stenosis (≥90%) of non-infarct arteries, and post-pPCI TIMI flow < 3 in the infarct-related artery. Detailed inclusion and exclusion criteria are provided in the Supplementary Table S1. Additional exclusions for analysis were death, cardiopulmonary resuscitation, acute heart failure, administration of inotropic therapy, use of mechanical circulatory support, hypertensive crisis, implantation of a temporary or permanent pacemaker, ICD, CRT-P, or CRT-D, recurrent myocardial infarction (including type IVa and IVb), cardiac surgery, or withdrawal of informed consent. Only patients with plasma samples available at all five planned time points were included in the biomarker analysis. The flow of patients through the study is shown in Supplementary Figure S1.

All participants provided written informed consent prior to enrollment.

### 2.2. Study Procedures

Venous blood samples were obtained at five time points: at hospital admission (0 h), and then at 12 ± 4 h, 24 ± 4 h, 48 ± 8 h, and 96 ± 8 h after the end of the pPCI. The admission sample was taken in the emergency triage unit, before coronary angiography, within approximately 15 min of arrival. Blood was collected into EDTA tubes and centrifuged immediately upon draw (2000× *g*, 10 min, room temperature). Plasma was then aliquoted into 500 µL microcentrifuge vials and stored at −20 °C until further analysis. Repeated freeze–thaw cycles of the samples were avoided. Demographic, clinical, and procedural data were recorded from electronic medical records, including relevant medical history, laboratory values, echocardiography parameters, and procedural details. LVEF was assessed by transthoracic echocardiography, performed as part of routine clinical care in the emergency triage unit before pPCI and before study enrollment, by experienced cardiologists using visual (semi-quantitative) estimation. The echocardiographers were not aware of the biomarker results, which were measured later. An LVEF < 40% was used as the usual cut-off for reduced LVEF.

### 2.3. Biomarker Analysis

Plasma levels of sST2, Gal-3, MR-proADM, and GDF-15 were determined by ELISA at the Institute of Medical and Clinical Biochemistry, Faculty of Medicine, University of Belgrade, Belgrade, Serbia, using human ELISA kits from ELK Biotechnology, Wuhan, China (#ELK11195, #ELK2790, #ELK9597, and #ELK2756, respectively), strictly according to the manufacturer’s instructions. The assay principle is sandwich ELISA: plates pre-coated with an antibody specific to the measured biomarker were incubated with the plasma samples, followed by the addition of a biotin-conjugated antibody specific to the same biomarker and subsequently avidin-conjugated horseradish peroxidase. After incubation with the substrate, the enzyme-catalyzed reaction was stopped with a sulfuric acid solution, producing a color change from blue to yellow, with color intensity directly proportional to biomarker concentration. Absorbance (optical density, O.D.) was measured at 450 nm using an automated microplate reader (Tecan, Dorset, UK). Biomarker concentrations were calculated by comparing sample O.D. values with standards using a four-parameter logistic curve fit in GraphPad Prism v.8 software; standard curves and the measurement range for each biomarker are provided in the Supplementary Figures S2–S5. Patient samples were measured in single wells. According to the manufacturer, the intra-assay and inter-assay coefficients of variation are <8% and <10%, respectively. All kits for each biomarker were from the same lot, and all samples from one patient were analyzed on the same plate. Values below the lowest standard were kept in the analysis, and their number for each biomarker is given in the Supplementary Material. Samples were stored at −20 °C for up to approximately 6 months (Gal-3, MR-proADM and GDF-15) and 18 months (sST2), which is within the reported stability of these biomarkers [32,33,34,35]. The laboratory staff had no access to clinical data but knew the sampling time points. sST2 concentrations are expressed in ng/mL and Gal-3, MR-proADM, and GDF-15 concentrations in pg/mL. Plasma hs-cTnI levels were measured in the local laboratory using the Access hsTnI chemiluminescent assay on the UniCel DxI 600 system (Beckman Coulter, Brea, CA, USA) and served as a reference marker of myocardial injury. According to the manufacturer, the limit of detection is 2.3 pg/mL, and the sex-specific 99th percentile upper reference limits are 11.6 pg/mL for women and 19.8 pg/mL for men. Samples above the highest calibrator (approximately 27,027 pg/mL) were diluted 1:10, and the results were automatically corrected by the analyzer. Peak biomarker concentrations were defined, for each analyte and each patient, as the highest value observed across the five sampling time points during the acute phase (0–96 h).

### 2.4. Statistical Analysis

Data were analyzed using descriptive and inferential statistical methods. Descriptive statistics included measures of central tendency (mean and median), measures of variability (standard deviation and interquartile range), and absolute and relative frequencies. Biomarker concentrations are presented on the original scale as means with 95% confidence intervals (95% CI), while inferential analyses were performed using natural log-transformed values. Changes in the concentrations of the five biomarkers (sST2, Gal-3, MR-proADM, GDF-15, and hs-cTnI) over time were analyzed using repeated-measures General Linear Models (GLM), with time (0, 12, 24, 48, and 96 h) as the within-subject factor. Sphericity was assessed using Mauchly’s test, and Greenhouse-Geisser correction was applied when the sphericity assumption was violated. Separate models were performed according to symptom duration (≤120 vs. >120 min), left ventricular ejection fraction (LVEF; <40% vs. ≥40%), and whether the left anterior descending artery (LAD) was the infarct-related artery, with each of these variables entered as a between-subject factor in the corresponding model. All repeated-measures GLM analyses were unadjusted and did not include additional covariates. Subgroup analyses were considered exploratory and hypothesis-generating. The main effects of time and group, as well as the time × group interaction, were evaluated. Statistical significance was set at *p* < 0.05. Three individual biomarker measurements were missing and were replaced with the median value for the corresponding biomarker and time point for the repeated-measures GLM analyses. Pairwise comparisons between time points were adjusted using the Sidak method. All statistical analyses were performed using IBM SPSS Statistics for Windows, version 26.0.

## 3. Results

Of 46 enrolled patients, 16 were excluded from the biomarker analysis, 15 because of incomplete serial sampling due to early discharge and 1 because sST2 concentrations at 48 h and 96 h were below the limit of detection (Supplementary Figure S1). The analyses included 30 patients with STEMI, of whom 20 (66.7%) were male. The mean age was 57.9 ± 11.1 years (range 27–74 years), with no significant difference between men and women (56.3 ± 10.8 vs. 61.0 ± 11.7 years, *p* = 0.282). The median symptom duration was 120 min (IQR 60–180), with 70% of patients presenting within 2 h of symptom onset. Diabetes mellitus was present in 33.3% of patients, hypertension in 56.7%, hyperlipidemia in 43.3%, and 46.7% were former or active smokers. The mean LVEF was 40.8 ± 8.8%, with 43.3% of patients having an LVEF < 40%. The LAD was the infarct-related artery in 43.3% of cases. Mean serum creatinine was 78.8 ± 18.8 µmol/L, and mean eGFR was 91.0 ± 15.8 mL/min/1.73 m^2^. Compared with the included patients, excluded patients less often had LVEF < 40% (6.3% vs. 43.3%, *p* = 0.016), tended to have higher mean LVEF (46.2 ± 5.0% vs. 40.8 ± 8.8%, *p* = 0.053), and more often had hypertension (87.5% vs. 56.7%, *p* = 0.049), while other characteristics did not differ significantly. Detailed demographic and clinical characteristics of all enrolled patients are presented in Table 1.

**Figure 1:**
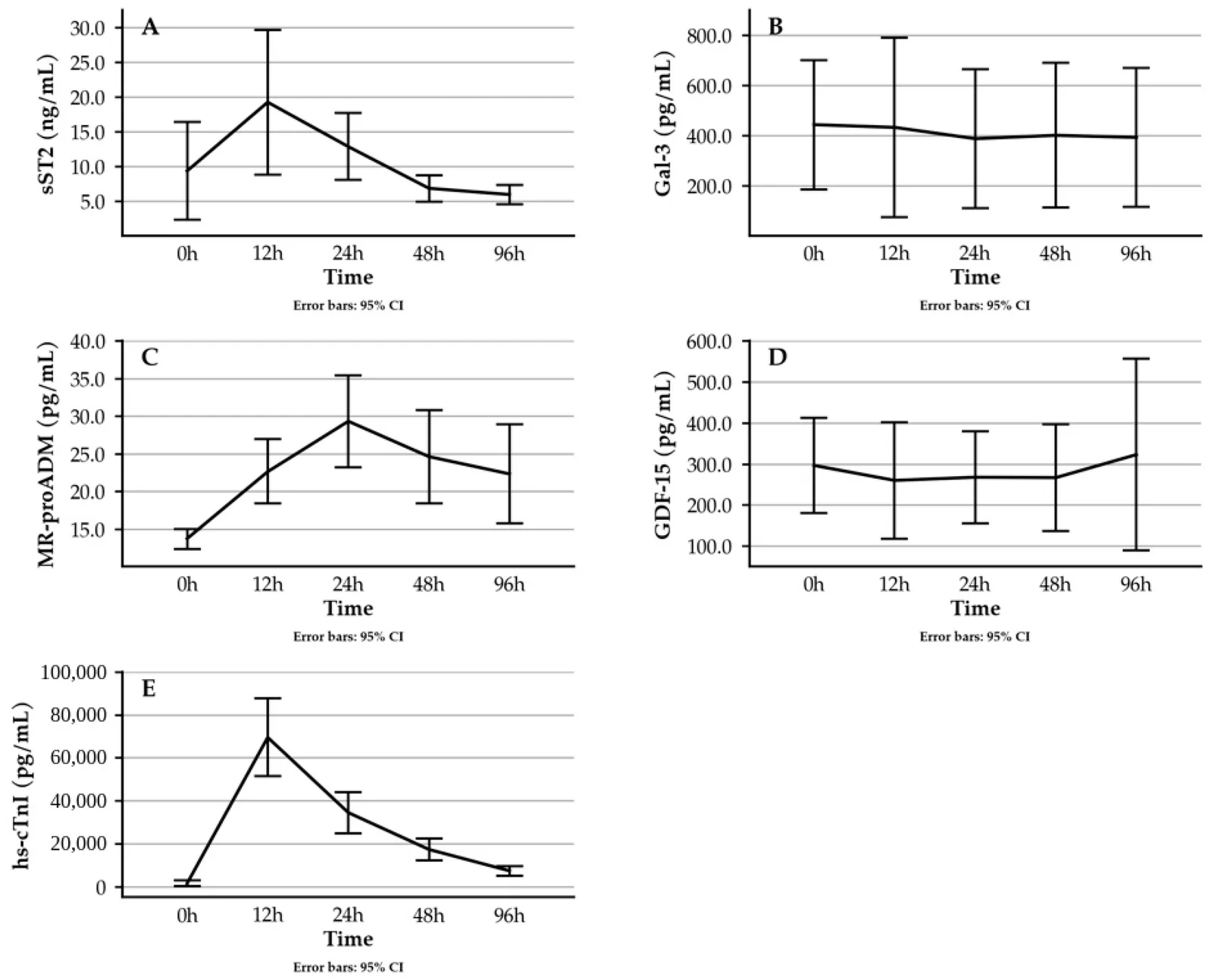
Changes in biomarker concentrations over time in patients with STEMI: (**A**) sST2, (**B**) Gal-3, (**C**) MR-proADM, (**D**) GDF-15, and (**E**) hs-cTnI. Values are presented as mean (95% CI).

Changes in the concentrations of all five biomarkers over the 96 h follow-up period are shown in Figure 1. sST2 increased during the first 12 h and subsequently decreased (*p* < 0.001), while MR-proADM reached its highest concentration at 24 h and declined thereafter, but remained above baseline at 96 h (*p* < 0.001). In contrast, no significant change over time was found for Gal-3 and GDF-15 (*p* = 0.260 and *p* = 0.416, respectively). hs-cTnI showed a marked increase from baseline to 12 h, followed by a progressive decline through 96 h (*p* < 0.001) (Figure 1). Sidak-adjusted pairwise comparisons were consistent with these overall temporal patterns. Detailed biomarker concentrations with 95% confidence intervals, absolute and percentage changes from baseline, overall time effects, and pairwise time-point contrasts are provided in Supplementary Tables S2 and S3.

Repeated-measures GLM analysis showed significant changes over time in sST2, MR-proADM, and hs-cTnI levels according to symptom duration (≤120 min, *n* = 21; >120 min, *n* = 9; all time effects, *p* < 0.001), whereas no significant temporal changes were observed for Gal-3 (*p* = 0.391) or GDF-15 (*p* = 0.211) (Table 2). sST2 levels increased markedly from baseline to 12 h in both groups and subsequently declined through 96 h. MR-proADM levels also increased during the early follow-up period, reaching their highest values at 24 h, while hs-cTnI levels reached their highest values at 12 h and decreased thereafter. No significant overall differences between patients with symptom duration ≤ 120 min and those with symptom duration > 120 min were observed for any of the five biomarkers (*p* > 0.050) (Table 2). Furthermore, none of the time-by-group interactions reached statistical significance (*p* > 0.050) (Table 2).

When patients were stratified according to LVEF (<40%, *n* = 13; ≥40%, *n* = 17), significant changes over time were observed in sST2, MR-proADM, and hs-cTnI levels (all time effects, *p* < 0.001), whereas no significant temporal changes were found for Gal-3 (*p* = 0.347) or GDF-15 (*p* = 0.352) (Table 3). Significant overall differences between LVEF groups were observed for sST2 (*p* = 0.009), MR-proADM (*p* = 0.019), and hs-cTnI (*p* = 0.002) (Table 3), with higher concentrations in patients with LVEF <40% compared with those with LVEF ≥ 40%. No significant group differences were found for Gal-3 (*p* = 0.869) or GDF-15 (*p* = 0.472) (Table 3). No significant time-by-group interactions were observed for any of the five biomarkers (all *p* > 0.050) (Table 3).

According to infarct-related artery status (LAD, *n* = 13; non-LAD, *n* = 17), significant temporal changes were observed in sST2, MR-proADM, and hs-cTnI (all *p* < 0.001), while Gal-3 (*p* = 0.325) and GDF-15 (*p* = 0.430) showed no significant temporal changes (Table 4). sST2 and hs-cTnI concentrations increased from baseline to 12 h and subsequently declined through 96 h in both groups. MR-proADM concentrations increased during the early follow-up period, reaching their highest mean values at 24 h, followed by a gradual decline. Among the five biomarkers, significant overall differences between groups were observed only for sST2 (*p* = 0.005) and hs-cTnI (*p* = 0.007) (Table 4), with higher concentrations in patients with LAD as the infarct-related artery. No significant group differences were observed for Gal-3 (*p* = 0.670), MR-proADM (*p* = 0.142), or GDF-15 (*p* = 0.418) (Table 4). No significant time-by-group interactions were observed for any of the five biomarkers (*p* > 0.050) (Table 4).

In a post hoc sensitivity analysis excluding patients with hs-cTnI 0 h > 1000 pg/mL (*n* = 24), the overall temporal patterns remained consistent. Significant time-dependent changes persisted for sST2 (*p* < 0.001), MR-proADM (*p* < 0.001) and hs-cTnI (*p* < 0.001), whereas Gal-3 and GDF-15 showed no significant temporal changes (*p* = 0.736 and *p* = 0.763, respectively).

## 4. Discussion

A multimarker approach to risk stratification after STEMI treated with primary PCI increasingly incorporates biomarkers that reflect distinct pathophysiological pathways: neurohormonal stress (MR-proADM), mechanical stress and myocardial fibrosis (sST2), systemic stress and inflammation (GDF-15), and profibrotic/remodeling activity (Gal-3). All four have shown independent prognostic value for mortality and the development of heart failure after acute myocardial infarction in previous studies. High baseline sST2 level, independent of NT-proBNP, is a strong predictor of cardiovascular mortality and heart failure after STEMI [36,37], while elevated MR-proADM level at admission is an independent predictor of both short- and long-term mortality and heart failure [38,39]. Gal-3 has been described as a molecule positioned at the intersection of the acute infarct phase, inflammation, and subsequent myocardial remodeling [40,41], and GDF-15 has been shown to be a strong predictor of 30-day mortality when measured at 0 h, 12 h, and 24 h after STEMI [42].

However, despite this consistent prognostic value, the kinetics of these biomarkers in the acute phase of infarction remain far less well characterized than those of classical necrosis markers (troponin, CK-MB), whose peaks and rise/decline dynamics are well defined and standardized by established sampling protocols. This represents a significant methodological gap: in a large proportion of studies, each biomarker is measured only once (usually on admission), without knowing whether that time point represents the peak, the rising limb, or an already declining concentration, which directly affects the interpretation of its prognostic strength. A study that is particularly relevant in this context directly compared, at five identical time points, the kinetics of sST2 in acute ischemic stroke and STEMI treated with thrombectomy and pPCI respectively, specifically in order to establish the kinetics of its release and its association with adverse outcomes in both populations before considering sST2 as a therapeutic target [43]. Conceptually, this is the closest existing design to what would be required for the biomarkers we examined, and it directly motivated the five-time-point protocol used in the present study.

Our findings partly confirm and partly extend this literature. sST2 and MR-proADM showed statistically significant temporal change, with the highest measured concentrations of sST2 at 12 h and of MR-proADM later, at 24 h, whereas no significant change over time was found for Gal-3 and GDF-15 during the 96 h observation period. The sST2 kinetic profile we observed is consistent with prior reports describing a rise beginning around 3 h after STEMI onset and a peak near 12 h [44], and is closely in line with the HIBISCUS-STEMI cohort, in which sST2 peaked at 24 h after admission and elevated 24 h sST2 levels were independently associated with 12-month MACE [43]. The higher sST2 concentrations in patients with reduced LVEF and LAD infarction are in line with the established link between sST2 and mechanical myocardial strain and left ventricular functional impairment [45], and are consistent with a serial proteomic analysis in STEMI patients (baseline, 6 h, 24 h, and 96 h sampling) in which sST2, alongside IL-6, showed the strongest association among 131 circulating proteins with both infarct size and LVEF on CMR [46], in keeping with data showing sST2 to correlate with infarct size, no-reflow, and subsequent heart-failure risk [39,47]. However, peak hs-cTnI was also higher in patients with LVEF < 40% (median 83,045 vs. 41,938 pg/mL, *p* = 0.045), so these differences may largely reflect larger infarct size. In addition, the shape of the sST2 curve did not differ significantly between subgroups.

MR-proADM showed a distinct, delayed pattern, with the highest measured concentrations at 24 h and values still above baseline at 96 h. To our knowledge, serial MR-proADM measurements in uncomplicated STEMI treated with contemporary pPCI have rarely been reported, since previous work largely relied on single admission measurements or on sampling restricted to complicated presentations, such as cardiogenic shock at 24 h [48] or heart-failure onset at a median of 3 days post-MI in the OPTIMAAL cohort [38]. The higher MR-proADM concentrations in patients with reduced LVEF may suggest that this delayed rise reflects the degree of early hemodynamic compromise rather than the acute necrotic process itself. In a prospective study of 80 patients with myocardial infarction treated with PCI, Węgiel et al. found that higher MR-proADM concentrations in the acute phase predicted adverse left ventricular remodeling at 6 months [49]. Given the delayed rise in MR-proADM in our study, the timing of blood sampling may affect its prognostic value, which should be considered in future studies.

In contrast, Gal-3 and GDF-15 concentrations did not change significantly over the 96 h window. This should not be interpreted as proof of biological stability, because it may also reflect the small sample size, assay variability, the time between samples, and the fact that a considerable number of Gal-3 (22 of 149) and GDF-15 (66 of 149) measurements were below the lowest standard of the assay. The absence of significant change in Gal-3 is in line with reports finding no significant difference in concentration across days 1, 5, and 30 after pPCI [50], even though other groups have described an early rise within 4–6 h [40,51]; this discrepancy across studies likely reflects differences in assay methodology, reperfusion timing, or the possibility that Gal-3 in the first 96 h predominantly reflects background macrophage/fibrotic activity rather than an infarct-triggered acute response, becoming more dynamic only during the subacute-to-chronic remodeling phase. For GDF-15, this is compatible with observations showing only a weak, gradual rise with limited intra-individual variability once the initial stress-induced elevation has occurred [52], and with studies describing a rapid early increase within minutes to a few hours, followed by a prolonged plateau rather than a sharp, transient peak [53,54]. This may suggest that GDF-15 behaves less like a marker of acute necrosis and more like a sustained stress-response cytokine, whose trajectory becomes informative only over a much longer horizon, as suggested by serial studies extending to weeks after the index event [52,55].

Taken together, our data suggest that a uniform sampling strategy may not be appropriate for this biomarker panel: sST2 and MR-proADM demonstrated distinct temporal patterns, with the highest observed concentrations occurring approximately 12 and 24 h after pPCI, respectively, while Gal-3 and GDF-15 may require either earlier sampling or a longer follow-up to show meaningful change related to fibrotic and reparative processes. These findings may be useful for the design of future studies. A single blood sample may give different sST2 and MR-proADM values depending on when it is taken, while Gal-3 and GDF-15 seem less dependent on sampling time within the first 96 h. Future prognostic studies should therefore clearly report the time of sampling in relation to reperfusion, consider 12 h (sST2) and 24 h (MR-proADM) as candidate sampling windows, and, if possible, include serial measurements. Such studies should also test whether sampling within these windows improves the prediction of adverse remodeling and clinical outcomes compared with sampling at admission.

## 5. Limitations

This study has several limitations. First, it was designed as an exploratory pilot study, and the small sample size (*n* = 30) limits statistical power, particularly for subgroup analyses, which should be regarded as hypothesis-generating. Second, 16 enrolled patients were excluded, mostly because of early discharge. Excluded patients less often had reduced LVEF, so the analyzed group may include more patients with left ventricular dysfunction, and the kinetic profiles may not fully apply to patients with preserved LVEF. Third, LVEF was assessed by visual estimation at presentation, before reperfusion, and therefore reflects acute ischemic dysfunction rather than final left ventricular function. Longitudinal imaging to assess left ventricular remodeling was not performed, and clinical outcomes were not evaluated. Fourth, sampling after pPCI was timed from the end of the procedure rather than from symptom onset, and exact sampling times, door-to-balloon times and symptom onset-to-reperfusion times were not recorded. Fifth, research-use ELISA kits were used; samples were measured in single wells, in-house assay precision was not determined, samples were stored at −20 °C, and a considerable number of MR-proADM, Gal-3 and GDF-15 measurements were below the lowest standard. Therefore, absolute concentrations cannot be directly compared with those measured by validated clinical assays, and our results should be interpreted mainly as relative changes within patients. Finally, this was a single-center study with strict exclusion criteria, which limits generalizability to more complicated STEMI presentations. Despite these limitations, the study provides serial data at five predefined time points in a homogeneous group of patients with uncomplicated STEMI, which may help in planning future, adequately powered prognostic studies.

## 6. Conclusions

Among the four biomarkers studied, sST2 and MR-proADM, but not Gal-3 or GDF-15, showed distinct kinetics within the first 96 h after STEMI treated with pPCI, with the highest measured concentrations at 12 and 24 h, respectively. In exploratory analyses, sST2 and MR-proADM concentrations were higher in patients with reduced LVEF, and sST2 concentrations in patients with LAD infarction. Larger, multicenter studies with extended follow-up and serial echocardiographic assessment are needed to confirm these kinetics and to determine whether sampling at these time points is prognostically informative.

## Figures and Tables

**Table 1:** Baseline demographic and clinical characteristics of patients with STEMI.

Variable	Included (*n* = 30)	Excluded (*n* = 16)	*p*
Sex, *n* (%)			1.000
Male	20 (66.7)	11 (68.8)	
Female	10 (33.3)	5 (31.3)	
Age (years), mean ± SD	57.9 ± 11.1	56.6 ± 11.7	0.556
Symptom duration (min), median (IQR)	120 (60–180)	105 (60–180)	0.907
Diabetes mellitus, *n* (%)	10 (33.3)	2 (12.5)	0.170
Hypertension, *n* (%)	17 (56.7)	14 (87.5)	0.049
Hyperlipidemia, *n* (%)	13 (43.3)	3 (18.8)	0.117
Former or active smoking, *n* (%)	14 (46.7)	8 (50.0)	1.000
Family history of cardiovascular disease, *n* (%)	11 (36.7)	8 (50.0)	0.531
LVEF (%), mean ± SD	40.8 ± 8.8	46.2 ± 5.0	0.053
LVEF < 40%, *n* (%)	13 (43.3)	1 (6.3)	0.016
Symptom duration >120 min, *n* (%)	9 (30.0)	7 (43.8)	0.517
LAD as infarct-related artery, *n* (%)	13 (43.3)	4 (25.0)	0.338
Serum creatinine (µmol/L), mean ± SD	78.8 ± 18.8	89.0 ± 38.3 *	0.547
eGFR (mL/min/1.73 m^2^), mean ± SD	91.0 ± 15.8	88.2 ± 26.2 *	0.800
Number of stents implanted, *n* (%)			0.877
One	23 (76.7)	12 (75.0)	
Two	6 (20.0)	3 (18.8)	
Three	1 (3.3)	1 (6.3)	

Values are presented as *n* (%), mean ± SD, or median (IQR), as appropriate. * *n* = 15 for serum creatinine and eGFR in the excluded group. Continuous variables were compared using the Mann–Whitney *U* test and categorical variables using Fisher’s exact test. eGFR, estimated glomerular filtration rate (CKD-EPI 2021); LAD, left anterior descending artery; LVEF, left ventricular ejection fraction.

**Table 2:** Changes in biomarker concentrations over time according to symptom duration.

Biomarker	Symptom Duration		Time *p*	Group *p*	Time × Group *p*
	≤120 min *n *= 21	>120 min *n* = 9			
sST2 (ng/mL)					
0 h	5.6 (4.7–6.4)	18.2 (−7.9–44.2)			
12 h	14.8 (9.5–20.1)	29.7 (−7.3–66.6)			
24 h	10.3 (7.0–13.7)	18.7 (2.9–34.5)	<0.001	0.248	0.252
48 h	6.1 (4.5–7.8)	8.3 (2.5–14.1)			
96 h	5.2 (3.9–6.5)	7.5 (3.4–11.5)			
Gal-3 (pg/mL)					
0 h	518.9 (147.0–890.8)	263.2 (212.8–313.7)			
12 h	506.8 (−12.8–1026.5)	255.1 (191.1–319.0)			
24 h	441.9 (39.0–844.7)	256.5 (165.7–347.2)	0.391	0.423	0.639
48 h	469.6 (51.6–887.5)	236.3 (161.2–311.4)			
96 h	450.2 (47.5–853.0)	253.6 (187.5–319.7)			
MR-proADM (pg/mL)					
0 h	12.8 (11.2–14.3)	15.8 (13.2–18.3)			
12 h	20.3 (15.2–25.4)	28.0 (20.2–35.9)			
24 h	27.6 (21.1–34.1)	33.3 (17.5–49.0)	<0.001	0.207	0.445
48 h	23.7 (15.6–31.8)	26.7 (15.8–37.5)			
96 h	20.0 (16.7–23.3)	27.7 (4.0–51.5)			
GDF-15 (pg/mL)					
0 h	280.2 (139.7–420.8)	333.4 (76.3–590.4)			
12 h	288.7 (84.2–493.2)	191.9 (102.9–280.9)			
24 h	282.2 (122.6–441.8)	232.9 (124.7–341.0)	0.211	0.639	0.076
48 h	303.3 (116.0–490.6)	180.4 (110.3–250.5)			
96 h	394.9 (58.9–731.0)	153.6 (84.7–222.6)			
hs-cTnI (pg/mL)					
0 h	590.5 (−92.0–1273.1)	3623.7 (−985.3–8232.6)			
12 h	67,366.7 (43,192.2–91,541.3)	74,236.7 (45,343.5–103,129.9)			
24 h	31,081.0 (20,741.3–41,420.8)	42,202.7 (17,464.0–66,941.4)	<0.001	0.094	0.074
48 h	14,697.2 (9943.8–19,450.6)	23,339.8 (9090.6–37,589.0)			
96 h	6514.4 (3885.5–9143.3)	9274.0 (4140.2–14,407.8)			

Values are presented as mean (95% CI). *p* values were obtained from repeated-measures GLM performed on natural log-transformed concentrations.

**Table 3:** Changes in biomarker concentrations over time according to LVEF.

Biomarker	LVEF		Time *p*	Group *p*	Time × Group *p*
	<40% *n* = 13	≥40% *n* = 17			
sST2 (ng/mL)					
0 h	14.8 (−2.2–31.8)	5.1 (4.2–6.1)			
12 h	30.7 (6.5–54.8)	10.5 (7.9–13.1)			
24 h	18.8 (8.1–29.4)	8.3 (5.8–10.8)	<0.001	0.009	0.499
48 h	9.2 (5.1–13.3)	5.0 (4.0–6.0)			
96 h	7.6 (4.6–10.5)	4.6 (3.6–5.6)			
Gal-3 (pg/mL)					
0 h	289.3 (247.8–330.8)	559.2 (92.1–1026.2)			
12 h	292.8 (240.0–345.7)	537.2 (−118.1–1192.5)			
24 h	290.3 (227.6–353.0)	459.6 (−48.9–968.1)	0.347	0.869	0.494
48 h	294.8 (245.0–344.6)	479.7 (−49.4–1008.8)			
96 h	286.6 (230.7–342.4)	471.3 (−36.6–979.0)			
MR-proADM (pg/mL)					
0 h	14.5 (12.7–16.4)	13.0 (10.9–15.1)			
12 h	29.1 (22.1–36.0)	17.7 (13.2–22.1)			
24 h	35.7 (23.7–47.8)	24.4 (18.8–30.0)	<0.001	0.019	0.267
48 h	27.7 (20.3–35.1)	22.2 (12.2–32.2)			
96 h	27.7 (12.8–42.7)	18.2 (14.1–22.3)			
GDF-15 (pg/mL)					
0 h	360.8 (190.3–531.3)	246.8 (75.9–417.6)			
12 h	248.4 (143.7–353.1)	268.4 (16.5–520.2)			
24 h	254.0 (156.0–352.0)	277.6 (82.1–473.1)	0.352	0.472	0.234
48 h	215.8 (147.4–284.2)	305.1 (70.4–539.9)			
96 h	265.5 (161.5–369.6)	366.2 (−58.2–790.5)			
hs-cTnI (pg/mL)					
0 h	1741.9 (−269.2–3753.0)	1315.9 (−742.0–3373.7)			
12 h	84,981.4 (62,153.3–107,809.5)	57,533.8 (30,165.2–84,902.4)			
24 h	45,196.8 (29,136.4–61,257.2)	26,174.6 (14,504.2–37,845.0)	<0.001	0.002	0.270
48 h	24,914.2 (15,748.7–34,079.7)	11,459.7 (6813.6–16,105.8)			
96 h	10,385.1 (6238.0–14,532.1)	5015.5 (2810.1–7220.9)			

Values are presented as mean (95% CI). *p* values were obtained from repeated-measures GLM performed on natural log-transformed concentrations.

**Table 4:** Changes in biomarker concentrations over time according to infarct-related artery status.

Biomarker	LAD		Time *p*	Group *p*	Time × Group *p*
	No *n* = 17	Yes *n* = 13			
sST2 (ng/mL)					
0 h	5.2 (4.3–6.1)	14.8 (−2.3–31.8)			
12 h	10.2 (7.6–12.8)	31.0 (7.0–55.1)			
24 h	7.6 (5.4–9.9)	19.6 (9.2–30.0)	<0.001	0.005	0.068
48 h	4.9 (3.9–5.9)	9.2 (5.2–13.3)			
96 h	4.7 (3.7–5.7)	7.5 (4.5–10.5)			
Gal-3 (pg/mL)					
0 h	571.4 (105.9–1036.9)	273.3 (245.1–301.4)			
12 h	554.1 (−99.2–1207.4)	270.8 (216.7–324.9)			
24 h	474.4 (−32.4–981.1)	271.0 (203.3–338.2)	0.325	0.670	0.784
48 h	499.1 (−27.8–1026.1)	269.4 (218.9–319.9)			
96 h	487.5 (−18.6–993.5)	265.4 (211.1–319.6)			
MR-proADM (pg/mL)					
0 h	13.4 (11.2–15.6)	14.0 (12.3–15.7)			
12 h	20.2 (13.7–26.6)	25.8 (20.2–31.3)			
24 h	27.3 (19.3–35.2)	32.0 (21.4–42.5)	<0.001	0.142	0.564
48 h	23.6 (13.4–33.7)	25.9 (18.6–33.2)			
96 h	18.8 (14.6–23.0)	27.0 (11.8–42.1)			
GDF-15 (pg/mL)					
0 h	341.7 (140.3–543.0)	236.7 (143.8–329.5)			
12 h	313.8 (61.2–566.3)	189.0 (105.5–272.6)			
24 h	311.4 (115.4–507.3)	209.8 (125.7–294.0)	0.430	0.418	0.953
48 h	323.2 (90.6–555.8)	192.1 (125.6–258.7)			
96 h	407.1 (−13.6–827.8)	212.0 (110.8–313.2)			
hs-cTnI (pg/mL)					
0 h	1331.7 (−723.9–3387.2)	1721.3 (−295.8–3738.3)			
12 h	57,977.4 (31,814.2–84,140.6)	84,401.2 (58,860.4–109,942.0)			
24 h	26,506.7 (15,979.9–37,033.5)	44,762.5 (27,124.6–62,400.3)	<0.001	0.007	0.281
48 h	12,411.7 (7583.2–17,240.2)	23,669.3 (14,026.0–33,312.6)			
96 h	5340.8 (3170.4–7511.2)	9959.6 (5582.3–14,337.0)			

Values are presented as mean (95% CI). *p* values were obtained from repeated-measures GLM performed on natural log-transformed concentrations.

## Data Availability

The data presented in this study are available on request from the corresponding author.

## Supplementary Materials

The following supporting information can be downloaded at: https://www.mdpi.com/article/10.3390/cells15201868/s1, Supplementary Table S1: Inclusion and Exclusion Criteria; Supplementary Table S2: Biomarker concentrations and changes from baseline during the 96 h follow-up; Supplementary Table S3: Pairwise time-point contrasts; Supplementary Figure S1: Flow of patients through the study; Supplementary Figure S2: Four-parameter logistic curve for sST2; Supplementary Figure S3: Four-parameter logistic curve for Gal-3; Supplementary Figure S4: Four-parameter logistic curve for MR-proADM; Supplementary Figure S5: Four-parameter logistic curve for GDF-15.

## Abbreviations

The following abbreviations are used in this manuscript:

STEMI	ST-segment elevation myocardial infarction
pPCI	Primary percutaneous coronary intervention
sST2	Soluble suppression of tumorigenicity-2
Gal-3	Galectin-3
MR-proADM	Mid-regional pro-adrenomedullin
GDF-15	Growth differentiation factor 15
hs-cTnI	High-sensitivity cardiac troponin I
ELISA	Enzyme-linked immunosorbent assay
LVEF	Left ventricular ejection fraction
LAD	Left anterior descending artery
MI	Myocardial infarction
ST2L	Transmembrane (ligand-binding) isoform of ST2
IL-33	Interleukin-33
HFrEF	Heart failure with reduced ejection fraction
HFpEF	Heart failure with preserved ejection fraction
BNP	B-type natriuretic peptide
NT-proBNP	N-terminal pro-B-type natriuretic peptide
TIMI	Thrombolysis in myocardial infarction
ICD	Implantable cardioverter-defibrillator
CRT-P	Cardiac resynchronization therapy-pacemaker
CRT-D	Cardiac resynchronization therapy-defibrillator
EDTA	Ethylenediaminetetraacetic acid
GLM	General linear model
CI	Confidence interval
IQR	Interquartile range
eGFR	Estimated glomerular filtration rate
CKD-EPI	Chronic Kidney Disease Epidemiology Collaboration
CK-MB	Creatine kinase-MB
IL-6	Interleukin-6
CMR	Cardiac magnetic resonance
