# Supplementary material for: Temporal Dynamics of Emerging Cardiovascular Biomarkers in the Early Phase After Acute ST-Segment Elevation Myocardial Infarction: A Prospective Single-Center Pilot Study
Source: Cells. 2026 Oct 14;15(20):1868. doi: 10.3390/cells15201868 (PMC13702699; doi:10.3390/cells15201868)
Supplement: Supplementary file 1 [file cells-15-01868-s001.zip › cells-4539193-supplementary.pdf]

SUPPLEMENTARY MATERIAL

Study Population

The predefined inclusion and exclusion criteria applied for patient selection are summarized in Supplementary Table S1. The flow of patients through the study is shown in Supplementary Figure S1.

Supplementary Table S1. Inclusion and exclusion criteria.

**Inclusion criteria**
Age 18-75 years
Acute ST-segment elevation myocardial infarction (STEMI) as the first manifestation of coronary artery disease, treated with primary percutaneous coronary intervention (pPCI) within a maximum of 6 hours from symptom onset
Willingness to participate in clinical study
**Exclusion criteria**
Previously known coronary artery disease, including previous acute myocardial infarction, unstable angina, stable coronary artery disease verified by non-invasive or invasive testing, or previous myocardial revascularization (percutaneous or surgical)
Previously known heart failure
Previously known atrial fibrillation
Previously known ventricular arrhythmia, except ventricular extrasystoles
Previously known cardiac conduction disorder requiring pacing
Previously known pulmonary thromboembolism
Previously known valvular heart disease, except mild or moderate mitral regurgitation, mild aortic stenosis or regurgitation, and mild or moderate tricuspid or pulmonary regurgitation
Previously known cardiomyopathy (hypertrophic, dilated, non-dilated left ventricular cardiomyopathy, arrhythmogenic right ventricular dysplasia, or restrictive cardiomyopathy)
Previously known myocarditis or pericarditis
Previous pacemaker implantation
Previous cardiac surgery other than the surgical myocardial revascularization
Chronic kidney disease, grade IV or V
Liver fibrosis or cirrhosis
Pulmonary fibrosis
Active malignancy (less than 5 years from surgical treatment), except non-melanoma skin cancer
Previous chemotherapy or radiotherapy
Infection requiring systemic treatment
Chronic inflammatory disease
Substance use disorder
Body mass index ≥ 35 kg/m^2
Recanalized infarct-related artery before pPCI
Total occlusion or critical stenosis ($\geq 90\%$) of a non-infarct-related artery
Coronary flow in the infarct-related artery <TIMI 3 after pPCI

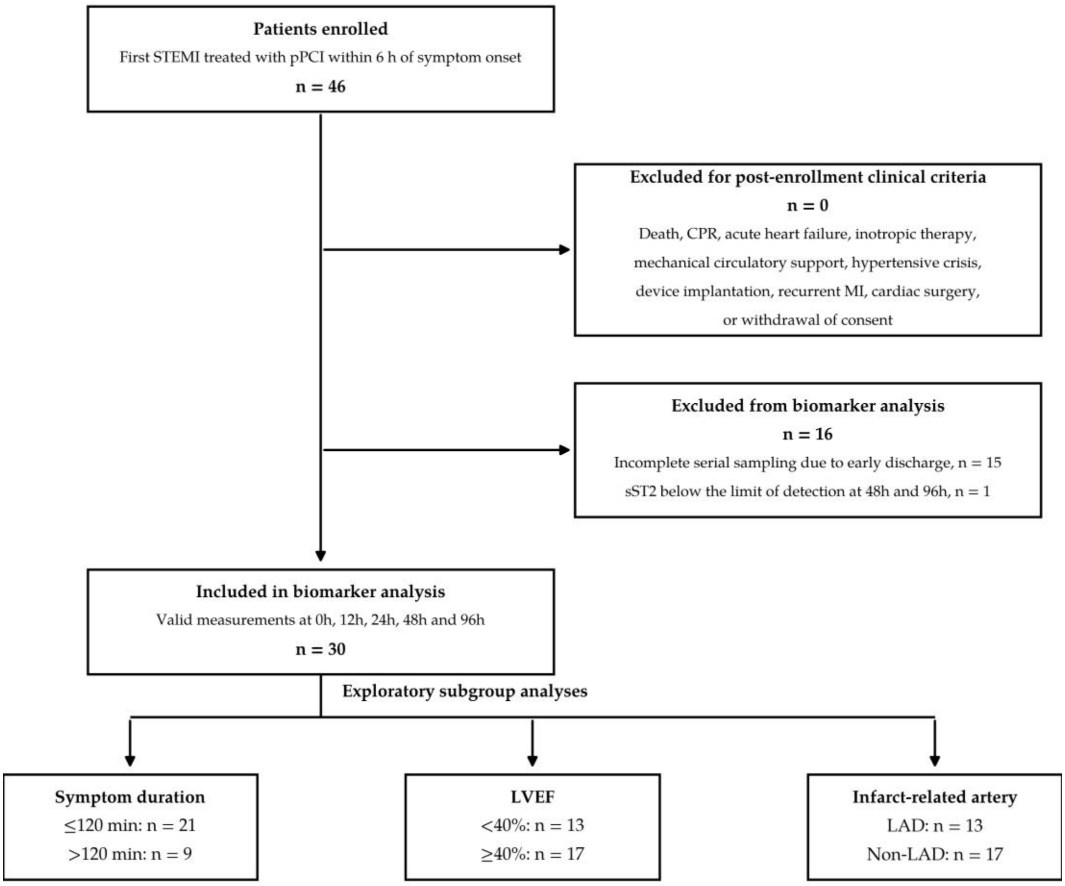

Supplementary Figure S1. Flow of patients through the study. CPR, cardiopulmonary resuscitation; LAD, left anterior descending artery; LVEF, left ventricular ejection fraction; MI, myocardial infarction; pPCI, primary percutaneous coronary intervention; sST2, soluble suppression of tumorigenicity-2; STEMI, ST-segment elevation myocardial infarction.

Biomarker Analysis

The standard curve for each measured biomarker was constructed using GraphPad Prism, based on the 7 standards, and then used for interpolation of unknown sample concentrations (95% confidence interval was used for the interpolation). Patient samples were measured in single wells. sST2 concentrations are expressed in ng/mL, and Gal-3, MR-proADM and GDF-15 concentrations in pg/mL.

The number of patient measurements below the lowest standard of the calibration curve was: sST2, 6 of 150 (<2.35 ng/mL; none below the analytical sensitivity), with one value slightly above the highest standard (154.3 ng/mL); Gal-3, 22 of 149 (<160 pg/mL; none below the analytical sensitivity); MR-proADM, 54 of 150 (<15.63 pg/mL), of which 7 were below the analytical sensitivity (9.17 pg/mL); GDF-15, 66 of 149 (<160 pg/mL), of which 6 were below the analytical sensitivity (53 pg/mL). These values were obtained by extrapolation of the standard curve and were kept in the analysis. Gal-3 and GDF-15 at 0h could not be measured in one patient because of insufficient sample volume.

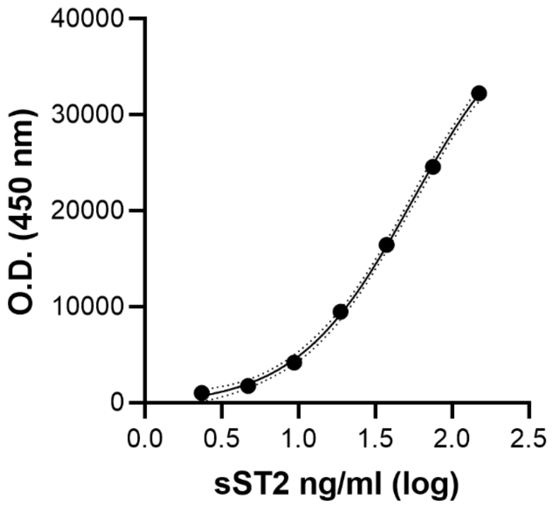

Supplementary Figure S2. Four-parameter logistic standard curve with 95% confidence bands for sST2, with O.D. (450 nm) on the y-axis and log concentration of sST2 (ng/mL) on the x-axis. The R squared for the goodness of fit is 0.9987. The reported detection range of the assay is 2.35–150 ng/mL, and the analytical sensitivity is 0.95 ng/mL.

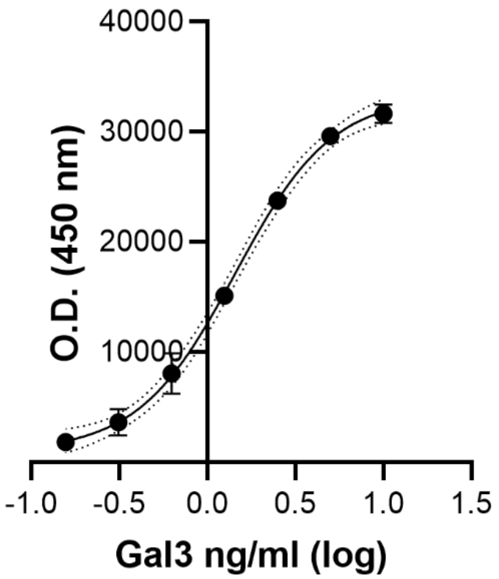

Supplementary Figure S3. Four-parameter logistic standard curve with 95% confidence bands for Gal-3, with O.D. (450 nm) on the y-axis and log concentration of Gal-3 (ng/mL) on the x-axis. The R squared for the goodness of fit is 0.9964. The reported detection range of the assay is 0.16–10 ng/mL (160–10,000 pg/mL), and the analytical sensitivity is 0.064 ng/mL (64 pg/mL). Gal-3 concentrations in the manuscript are expressed in pg/mL.

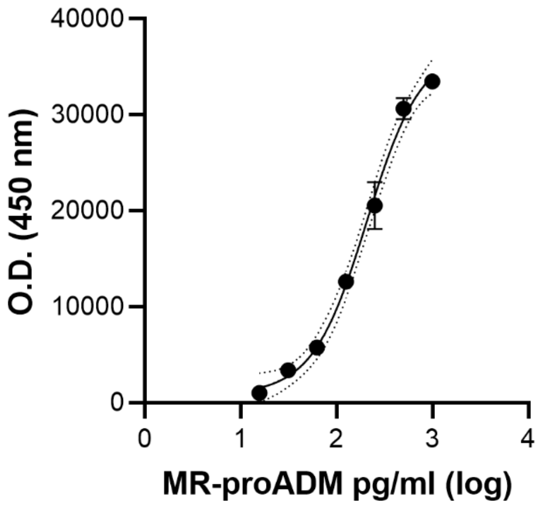

Supplementary Figure S4. Four-parameter logistic standard curve with 95% confidence bands for MR-proADM, with O.D. (450 nm) on the y-axis and log concentration of MR-proADM (pg/mL) on the x-axis. The R squared for the goodness of fit is 0.9932. The reported detection range of the assay is 15.63–1000 pg/mL, and the analytical sensitivity is 9.17 pg/mL.

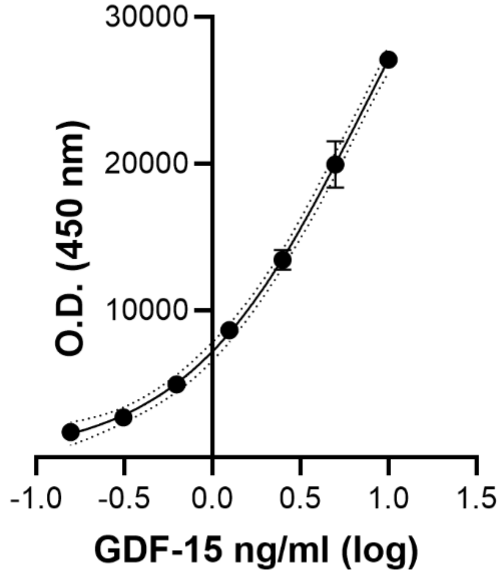

Supplementary Figure S5. Four-parameter logistic standard curve with 95% confidence bands for GDF-15, with O.D. (450 nm) on the y-axis and log concentration of GDF-15 (ng/mL) on the x-axis. The R squared for the goodness of fit is 0.9968. The reported detection range of the assay is 0.16–10 ng/mL (160–10,000 pg/mL), and the analytical sensitivity is 0.053 ng/mL (53 pg/mL). GDF-15 concentrations in the manuscript are expressed in pg/mL.

Biomarker Kinetics

Supplementary Table S2. Temporal changes in biomarker concentrations during the 96-hour follow-up.

Biomarker	Mean (95% CI)	Δ vs 0h	%Δ vs 0h	Overall p
**sST2 (ng/mL)**				
**0h**	9.3 (2.3-16.4)	–	–	
**12h**	19.2 (8.8-29.6)	+9.9	+105.8%	
**24h**	12.8 (8.0-17.7)	+3.5	+37.2%	**<0.001**
**48h**	6.8 (4.9-8.7)	−2.6	−27.4%	
**96h**	5.9 (4.5-7.3)	−3.4	−36.9%	
**Gal-3 (pg/mL)**				
**0h**	442.2 (184.7-699.8)	–	–	
**12h**	431.3 (74.2-788.4)	−10.9	−2.5%	
**24h**	386.2 (108.9-663.5)	−56.0	−12.7%	0.260
**48h**	399.6 (111.3-687.8)	−42.7	−9.6%	
**96h**	391.2 (114.2-668.2)	−51.0	−11.5%	
**MR-proADM (pg/mL)**				
**0h**	13.7 (12.3-15.0)	–	–	
**12h**	22.6 (18.4-26.9)	+8.9	+65.4%	
**24h**	29.3 (23.2-35.4)	+15.6	+114.4%	**<0.001**
**48h**	24.6 (18.4-30.8)	+10.9	+79.8%	
**96h**	22.3 (15.7-28.9)	+8.7	+63.3%	
**GDF-15 (pg/mL)**				
**0h**	296.2 (179.8-412.6)	–	–	
**12h**	259.7 (117.5-401.9)	−36.5	−12.3%	
**24h**	267.4 (154.9-379.9)	−28.8	−9.7%	0.416
**48h**	266.4 (135.8-397.0)	−29.8	−10.0%	
**96h**	322.5 (89.1-555.9)	+26.4	+8.9%	
**hs-cTnI (pg/mL)**				
**0h**	1500.5 (130.2-2870.8)	–	–	
**12h**	69427.7 (51344.8-87510.7)	+67927.2	+4527.0%	
**24h**	34417.5 (24763.3-44071.7)	+32917.1	+2193.8%	**<0.001**
**48h**	17290.0 (12194.3-22385.7)	+15789.5	+1052.3%	
**96h**	7342.3 (5072.1-9612.5)	+5841.8	+389.3%	

Values are presented as arithmetic mean (95% CI) on the original measurement scale. Δ represents the absolute change from the baseline (0h) mean concentration, and %Δ represents the percentage change in the mean concentration relative to baseline. Overall p values represent the effect of time obtained from repeated-measures GLM performed on natural log-transformed biomarker concentrations, with Greenhouse–Geisser correction applied when the sphericity assumption was violated.

Supplementary Table S3. Pairwise time-point contrasts for biomarker concentrations during the 96-hour follow-up.

Time-point contrast	MD (ln scale)	95% CI	Sidak-adjusted p
**sST2**			
**0h vs 12h**	−0.721	−1.017 to −0.426	**<0.001**
**0h vs 24h**	−0.444	−0.726 to −0.162	**<0.001**
**0h vs 48h**	0.078	−0.183 to 0.339	0.991
**0h vs 96h**	0.193	−0.074 to 0.460	0.310
**12h vs 24h**	0.277	0.076 to 0.478	**0.002**
**12h vs 48h**	0.799	0.549 to 1.050	**<0.001**
**12h vs 96h**	0.914	0.570 to 1.259	**<0.001**
**24h vs 48h**	0.522	0.293 to 0.752	**<0.001**
**24h vs 96h**	0.638	0.310 to 0.965	**<0.001**
**48h vs 96h**	0.115	−0.077 to 0.307	0.565
**Gal-3**			
**0h vs 12h**	0.104	−0.163 to 0.371	0.941
**0h vs 24h**	0.150	−0.134 to 0.434	0.725
**0h vs 48h**	0.113	−0.168 to 0.394	0.929
**0h vs 96h**	0.114	−0.123 to 0.351	0.817
**12h vs 24h**	0.046	−0.097 to 0.189	0.984
**12h vs 48h**	0.009	−0.145 to 0.163	1.000
**12h vs 96h**	0.010	−0.139 to 0.159	1.000
**24h vs 48h**	−0.037	−0.183 to 0.110	0.998
**24h vs 96h**	−0.036	−0.204 to 0.132	0.999
**48h vs 96h**	0.001	−0.155 to 0.156	1.000
**MR-proADM**			
**0h vs 12h**	−0.417	−0.632 to −0.201	**<0.001**
**0h vs 24h**	−0.688	−0.950 to −0.426	**<0.001**
**0h vs 48h**	−0.482	−0.763 to −0.201	**<0.001**
**0h vs 96h**	−0.370	−0.663 to −0.078	**0.006**
**12h vs 24h**	−0.271	−0.512 to −0.031	**0.019**
**12h vs 48h**	−0.065	−0.350 to 0.219	0.999
**12h vs 96h**	0.046	−0.281 to 0.373	1.000
**24h vs 48h**	0.206	−0.011 to 0.423	0.072
**24h vs 96h**	0.318	0.044 to 0.592	**0.015**
**48h vs 96h**	0.112	−0.069 to 0.292	0.524
**GDF-15**			
**0h vs 12h**	0.229	−0.236 to 0.695	0.795
**0h vs 24h**	0.080	−0.377 to 0.537	1.000
**0h vs 48h**	0.102	−0.334 to 0.538	0.999
**0h vs 96h**	0.108	−0.322 to 0.538	0.998
**12h vs 24h**	−0.149	−0.433 to 0.135	0.731
**12h vs 48h**	−0.127	−0.415 to 0.161	0.882
**12h vs 96h**	−0.121	−0.436 to 0.194	0.946
**24h vs 48h**	0.022	−0.167 to 0.211	1.000
**24h vs 96h**	0.028	−0.322 to 0.378	1.000
**48h vs 96h**	0.006	−0.298 to 0.310	1.000
**hs-cTnI**			
**0h vs 12h**	−5.738	−6.851 to −4.626	**<0.001**
**0h vs 24h**	−5.041	−6.174 to −3.907	**<0.001**
**0h vs 48h**	−4.294	−5.375 to −3.213	**<0.001**
**0h vs 96h**	−3.364	−4.466 to −2.262	**<0.001**
**12h vs 24h**	0.698	0.545 to 0.851	**<0.001**
**12h vs 48h**	1.445	1.219 to 1.671	**<0.001**
**12h vs 96h**	2.375	2.115 to 2.634	**<0.001**
**24h vs 48h**	0.747	0.576 to 0.918	**<0.001**
**24h vs 96h**	1.677	1.429 to 1.924	**<0.001**
**48h vs 96h**	0.930	0.672 to 1.187	**<0.001**

MD, estimated mean difference. Estimated mean differences and 95% confidence intervals are presented on the natural log scale. Pairwise comparisons were derived from the repeated-measures GLM performed on natural log-transformed biomarker concentrations and were adjusted for multiple comparisons using the Sidak method. Contrasts were calculated as the first time point minus the second time point; therefore, a negative MD indicates a higher estimated value at the second time point.
